# Serological study of bovine herpes virus type 1 in dairy herds of Hamedan province, Iran

**Published:** 2013

**Authors:** Aliasghar Bahari, Jamal Gharekhani, Masoumeh Zandieh, Ali Sadeghi-Nasab, Hesameddin Akbarein, Ahmad Karimi-Makhsous, Morteza Yavari

**Affiliations:** 1 *Department of Clinical Sciences, Faculty of Paraveterinary Sciences, Bu-Ali Sina University, Hamedan, Iran; *; 2 *Iranian Veterinary Organization, Hamedan, Iran; *; 3 * Department of Food HygieneandQuality Control, Faculty of Veterinary Medicine, University of Tehran, Tehran, Iran.*

**Keywords:** BHV-1, Cattle, ELISA, Hamedan, Iran

## Abstract

A cross-sectional study with a random cluster sampling design was carried out to estimate the seroprevalence of bovine herpesvirus type 1 (BHV-1) in non-vaccinated dairy herds in Hamedan province, west of Iran. Simple random sampling was used for selection of cattle in each herd. Informative data about each herd and selected animals were recorded by the farm manager in a provided questionnaire. Blood samples were collected from 492 animals in 41 industrial herds. A commercial indirect ELISA test was used to determine the seropositivity against BHV-1. The individual and herd seroprevalence for BHV-1 were 58.74% and 82.93%, respectively. The intra-herd prevalences were ranged from 16.70% to 100%. Geographical characteristics of Hamedan province may explain the high sero-prevalence rates found in this study compared to those of others obtained from different parts of the country. The proportion of seropositive cows were increased with age (*p* <0.05). Animals from large and moderate sized herds had higher odds of seropositivity than those of small size herds. These findings could be related to the presence of a considerable number of BHV-1 carriers in this region. The high herd and animal prevalence found in the present study suggested necessity of implementing an intensive control program for reducing BHV-1 infection rates.

## Introduction

Infectious bovine rhinotracheitis/infectious pustular vulvovaginitis (IBR/IPV), caused by bovine herpes virus type 1 (BHV-1), is an important disease of domestic and wild cattle.^1^ Clinical signs of BHV-1 infection include symptoms of inflammatory reactions in both respiratory and genital tracts, and abortion. A systemic disease affecting visceral organs may develop in young calves.^2^ BHV-1 is a member of genus *Varicellovirus *in the subfamily *Alphaherpesvirinae, *which belongs to the *Herpesviridae *family. However, there is only one antigenic serotype of BHV-1 by conventional serological assays.^3^ Two subtypes of BHV-1.1 (respiratory subtype) and BHV-1.2 (respiratory and genital subtype) have been described for this virus, based on restriction enzyme cleavage patterns of viral DNA.^1^

The BHV-1 infection is mainly transmitted via respiratory, ocular or genital secretions through direct contact between animals. However, the infection may also spread via fresh or frozen semen from infected bulls as well as contaminated equipments.^1^ Following a primary infection with a field isolate or vaccination with an attenuated strain, BHV-1 can become latent. The latent BHV-1 infection can become reactivated and the animal may shed the virus following stimuli, for example transport, parturition and glucocorticoid therapy.^4^

BHV-1 infections can be diagnosed by serological tests that detect the virus, viral antigen or specific antibody and by nucleic acid-based tests that detect genomic DNA, nucleic acid hybridization and sequencing. The immune response to primary BHV-1 infection in experimentally infected cattle is characterized by the development of specific IgM and IgG antibodies at 7 days post inoculation. Bovine herpes virus type 1 infected cows are mainly detected by the presence of specific antibodies to the virus after acute phase and during latency. Both virus neutralization (VN) test and ELISA have been employed for detecting antibodies against BHV-1. The ELISA is a specific, sensitive and practical test for detection of antibodies and has advantages over the VN test.^5^^,^^6^ Moreover, several types of BHV-1 ELISA tests are commercially available and some of them can be used in conjugation with marker vaccines to detect infected cattle in vaccinated populations.^6^^-^^8^

The detection of latent BHV-l infection in cattle is important for control programs and trade activities in dairy farms. Several studies have already demonstrated large different seroprevalence from 22.68% in the south^   9 ^ to 48.90% in the northwest of Iran.^10^ There is no published data on BHV-1 prevalence in dairy herds located in western provinces of Iran. Therefore, the main purpose of the present study was to determine the seroprevalence of BHV-1 infection in dairy cattle herds of Hamedan province, Iran.

## Materials and Methods

**Study design. **A cross-sectional study, with a random cluster sampling design, carried out in 41 industrial dairy herds of Hamedan province (situated in the middle of western Iran) between June and August 2009. The study area lies between longitudes 47˚ 34´ to 49˚ 36´ E and latitudes 33˚ 59´ to 35˚ 48´ N. Hamadan province is located in a temperate mountainous region with mean annual precipitation of 330 mm and mean annual temperature is 11.4 ˚C. The populations of cows in the tested farms were 50 to 660. Before sampling, the herds were divided to small (50-100 cows), medium (100-400 cows) and large (> 400 cows) units. No vaccination against BHV-1 was carried out in the farms; however, artificial insemination was applied in most of those herds. In each selected farm, individual cattle were chosen by random sampling method. The tested animals were divided to group A (between 6 months and 2 years), B (between 2 and 3 years), C (between 3 and 4 years) and D (more than 4 years), in order to study the association of age with sero-prevalence of the virus. To avoid false positive results due to potentially presence of maternal antibodies, calves younger than six months age were excluded from the study. 

**Serological test. **Four hundred ninety two blood samples were collected from coccygeal vein using plain vacutainer tubes, and transferred to the laboratory on ice. The samples were centrifuged at 2000 *g* for 10 min to obtain serum. Sera were stored in the labeled microtubes at -20 ˚C until analyzed. BHV-1 specific antibodies in the serum samples were detected using bovine rhinotracheitis virus (BHV-1) gE antibody ELISA test kit (HerdCheck^®^ IDEXX Laboratories, Inc., The Netherlands) according to manufacturer's instruction. Optic density (OD) was read at 650 nm in a multi-well plate reader (EL_X _800, BioTek Inc., Winooski, VT, USA). According to the manufacturer, the sensitivity and specificity of the kit was 98.00%. The Serum samples with OD ≥ 0.50 were considered as positive cases. 

**Data analysis. **Considering the reported specificity of the used kit (98.00%), herd with zero or one seropositive animals was defined as negative and herds with at least 2 seropositive animal as positive. Herd size, within-herd prevalence, overall individual and herd prevalence were calculated on the basis of the number of tested animals. Data analysis of Chi-Square and Fisher's exact test were carried out by SPSS version 16.0 (SPSS Inc., Chicago, IL, USA). A *p* value of less than 0.05 was considered statistically significant.

## Results

Seroprevalence of BHV-1 antibody in sampled animals was found to be 58.74% (289 of 492 sera) and according to presence of at least 2 seropositive animals in a herd, 34 herds (82.93%) were seropositive (Table 1).

**Table 1:** Seroprevalence of BHV-1 within examined herds and tested dairy cattle in Hamedan province, Iran (%).

**Herd size** *		**No. of herds**	**No. of sero-positive herds**	**No. of samples**	**No. of sero-positives ** †	**No. of sero-negatives** †
**Small**		18	14	216	104 (48.15) ^a^	112 (51.85) ^a^
**Medium **	18		15	216	139 (64.35) ^b^	77 (35.65) ^b^
**Large**		5	5	60	46 (76.67) ^ b^	14 (23.33) ^b^
**Total**		41	34	492	289 (58.74)	203 (41.26)

* Small: 50-100, medium: 100-400, large: > 400 dairy cows in herd.

† In each column, different letters show significant difference (*p *< 0.05).

The prevalence was ranged from 16.70 to 100% among seropositive herds in this study. All of 7 (17.10%) negative herds were from small and medium size herds (Table 1). Seroprevalence of BHV-1 within age groups was increased with age (Table 2). Based on age, the prevalence of seropositivity among group A was significantly different from other groups (*p* < 0.05). However, the prevalence rates between age groups of B, C and D showed no significant differences (Table 2). 

**Table 2:** Distribution of BHV-1 seroprevalence in dairy cattle with different ages of Hamedan province, Iran (%).

**Groups Age**		**No. of samples**	**No. of sero-positives ** *	**No. of sero-negatives ** *
**A 6 months to 2 years**		55	21 (38.18) ^a^	34 (61.82) ^a^
**B**	**2 to 3 years**	113	60 (53.10) ^b^	53 (46.90) ^b^
**C**	**3 to 4 years**	147	93 (63.27) ^b^	54 (36.73) ^b^
**D**	**more than 4 years**	177	115 (64.97) ^b^	62 (35.03) ^b^
	**Total**	492	289 (58.74)	203 (41.26)

* In each column, different letters show significant difference (*p *< 0.05).

## Discussion

The seroprevalence of BHV-1 in tested herds (82.93%) and tested animals (58.74%) found in this cross-sectional study was more than previously reported studies. Serological studies on the dairy cattle of different parts of Iran demonstrated 22.68% infection in Shiraz,^9^ 30.39% in Kerman,^11^ 31.48% in Ahvaz,^12^ 46.68% in Chaharmahal and Bakhtiari province^13^ and 48.9% in Urmia.^10^ It is reported that farms located in colder and higher altitude area are more prone to have experienced BHV infections.^14^ The climate and/or dairy cattle practice density in regions with higher prevalence is almost similar to Hamedan province from which obtained results were also similar. On the other hand, industrial dairy herd farming is not a common practice in low altitude areas of Iran which are often tropical and dry. The geographical variations may explain the differences between our results and low prevalence areas such as Shiraz, Kerman and Ahvaz. Infection prevalences in small herds are significantly less than moderate and large ones (Table 1). In our opinion, beside to lower natural exposure in small herds, biosecurity programs might be better implemented than moderate and large herds. The herd and animal BHV-1 prevalence in the current study did not differ greatly with those reported from countries that have no control program for BHV-1 infection.

The prevalence rates were 84.00% for dairy herds and 35.00% for dairy cows in Belgium,^15^ 50.0% in Germany,^16^ 80% for Hungary^17^ before start of their eradication programs and 61.00% in unvaccinated dairy herds of Italy.^18^ In 107 non-vaccinated herds in south west of England, mean seroprevalence of cattle and herds have been detected 42.50% and 43.00%, respectively.^14^ Considering herd size and management systems of dairy herds in each region or country, all the mentioned studies reported a moderate to high prevalence of infection prior to beginning an eradication program which may reflect the latency ability of the virus.

All breeds of cattle at any age are susceptible but the disease occurs most commonly in animals over 6 months old, probably because of their greater exposure (e.g. nasal exudate and coughed-up droplets, genital secretions, semen, fetal fluids and tissues and etc.) to the infective agent and loss maternal immunity.^19^ There was a positive relationship between age and proportion of seropositive cows in the present study. Similar findings were also reported by Boelaert *et al*.^15   ^ and Solis-Calderon *et al*.^20^ Since vaccination against BHV-1 was not practiced in the tested dairy herds, seropositivity was probably due to their naturally greater exposure to the virus. This fact is clearly demonstrated by significant difference between group A and other age groups (Table 2). Antibodies against BHV-1 persist throughout the animal's life after a natural infection.^1^ Moreover, the older animals are more likely to be exposed to natural sources of infection.^21^ Badiei *et al*. did not find any difference in the number of seropositive cows between age groups.^9^ They explained that their unexpectedly findings might be due to difference in herd size, cow rearing systems and animal keeping conditions.

Serologically positive cows which are latent carriers have the key roles in reserving and transmission of BHV-1 to susceptible animals.^22^ In agreement with Boelaert *et al*. and Solis-Calderon *et al*. the prevalence rate higher than 38.00% (Table 1) may indicate the naturally circulation of BHV-1 among animals.^15^^,^^20^ Moreover, the largest herds and older animals had highest seropositivity (Tables 1 and 2). This may show a high number of BHV-1 carriers in this herds. 

The method of isolation and slaughter of seropositive animals based on Danish and Swedish IBR control programs is inefficient for eradication of infection in countries with large herds or high seroprevalence of BHV-1. Several European countries have initiated control programs for eradication purposes based on using inactivated or live attenuated marker vaccines but no vaccine is able to prevent the infection and the establishment of latency.^23^^,^^24^ The marker vaccines used together with a serological detection of glycoprotein E-specific antibodies, allow differentiation of naturally infected cows from vaccinated animals.^25^

In conclusion, the high prevalence in herds and animals found in this study suggested necessity of an intensive control program for reducing BHV-1 infection rates. Based on present findings, we recommend using marker vaccine and serologically differentiation of naturally infected cows from vaccinated animals for eradication of IBR/IPV. Planned biosecurity measures are needed to control the epidemiological risk of infection due to the presence of BHV-1 latent carriers.
